# *APOE* Genetic Polymorphism rs7412 T/T Genotype May Be a Risk Factor for Essential Hypertension among Hakka People in Southern China

**DOI:** 10.1155/2022/8145896

**Published:** 2022-09-14

**Authors:** Hui Rao, Heming Wu, Zhikang Yu, Qingyan Huang

**Affiliations:** ^1^Department of Laboratory Medicine, Meizhou People's Hospital (Huangtang Hospital), Meizhou Academy of Medical Sciences, Meizhou, China; ^2^Guangdong Provincial Key Laboratory of Precision Medicine and Clinical Translational Research of Hakka Population, Meizhou People's Hospital (Huangtang Hospital), Meizhou Academy of Medical Sciences, Meizhou, China; ^3^Guangdong Provincial Engineering and Technology Research Center for Molecular Diagnostics of Cardiovascular Diseases, Meizhou People's Hospital (Huangtang Hospital), Meizhou Academy of Medical Sciences, Meizhou, China; ^4^Center for Precision Medicine, Meizhou People's Hospital (Huangtang Hospital), Meizhou Academy of Medical Sciences, Meizhou, China

## Abstract

**Objective:**

One of the causes of hypertension is a genetic factor. The purpose of this study was to look at the relationship between apolipoprotein E (APOE) and methylenetetrahydrofolate reductase (MTHFR) polymorphisms and essential hypertension in the Hakka population.

**Methods:**

The study included 2,850 patients with hypertension and 2,034 controls. *APOE* rs429358, rs7412, and *MTHFR* rs1801133 were genotyped by polymerase chain reaction (PCR)-microarray. The differences in these polymorphisms between the two groups were analyzed.

**Results:**

The genotype and allele frequency of *APOE* and *MTHFR* polymorphisms did not differ significantly between hypertensive patients and controls. Patients with hypertension who were *APOE* rs429358C/C homozygous had higher TG, TC, LDL-C, and Apo-B levels, whereas patients with the T/T genotype had higher HDL-C levels. Patients with hypertension who were *APOE* rs7412T/T homozygous had higher TG and TC levels and lower LDL-C and Apo-B levels. Homocysteine (Hcy) levels in patients with *MTHFR* CC, CT, and TT genotypes were increased, while patients with the TT genotype and T allele had higher Hcy levels than those of patients with other genotypes and the C allele. The *APOE* rs7412T/T genotype in the co-dominant model (*APOE* rs7412T/T vs. C/C) (gender-, age-, smoking-, and drinking-adjusted OR 2.682, 95% CI, 1.072–6.710, *P*=0.035) was a significant risk factor for hypertension. The *APOE* rs429358 and *MTHFR* rs1801133 genotypes in co-dominant, dominant, and recessive models were not significant risk factors for hypertension.

**Conclusions:**

It supports that *APOE* polymorphisms are related to hypertension in the Hakka population. Specifically, the *APOE* rs7412T/T genotype may be a risk factor for hypertension.

## 1. Introduction

Hypertension is one of the leading preventable risk factors for certain diseases [1, 2]. Hypertension is a chronic disease characterized by elevated blood pressure in the arteries of the systemic circulation [3–5]. Hypertension is the most prevalent risk factor for some diseases worldwide, affecting 1.39 billion people worldwide [6]. It is predicted that, by 2025, there will be 1.56 billion hypertensive patients all over the world [7]. Between October 2012 and December 2015, 23.2% (about 244.5 million) of the Chinese adults had hypertension and another 41.3% (about 435.3 million) had prehypertension [8]. Although awareness, control, and treatment rates in China have significantly improved, the prevalence of hypertension continues to rise [8]. Hypertension has become a major disease burden in China.

The pathogenesis of hypertension remains unclear. In recent years, scholars have carried out in-depth studies on the regulatory mechanisms underlying the occurrence and development of hypertension, and there are many possible mechanisms, including macrophage polarization [9], gene regulation [10, 11], renin-angiotensin-aldosterone system and sympathetic nervous system activation [12], central nervous system dysfunction [13], and renal damage [14]. The etiology of hypertension involves a complex interplay of environmental and genetic factors [15]. There is a significant genetic predisposition to hypertension, with genetic factors accounting for 30% to 50% of hypertension risks [16]. With the development of molecular biology techniques, the genetic predisposition to hypertension has been studied, but it still needs to be further elucidated.

Lipid levels have been linked to the risk of hypertension. A study has shown that serum triglyceride levels are significantly related to the development of hypertension [17]. High levels of TC and LDL-C are related to hypertension [18]. In addition, apolipoprotein E (ApoE) polymorphisms are associated with plasma lipoproteins [19, 20]. ApoE is one of the important apolipoproteins in plasma that binds to lipids and ApoE receptors (including LDLR and VLDLR), participates in lipid metabolism, and regulates cholesterol balance [21, 22]. ApoE is encoded by the *APOE* gene. There are two common polymorphisms in the *APOE* gene: rs429358 (388T>C, Cys112Arg) and rs7412 (526C>T, Arg158Cys), which result in three major alleles (*ɛ*2(388T–526T), *ɛ*3(388T–526C), and *ɛ*4(388C–526C)) [22, 23].

The level of serum homocysteine (Hcy) is related to the incidence of hypertension [24]. Hcy is a sulfur-containing amino acid that can damage blood vessels [25]. Hyperhomocysteinemia is associated with the incidence of hypertension and significantly increases the risk of vascular disease [26, 27]. MTHFR is a key enzyme in Hcy metabolism [28]. MTHFR is encoded by the *MTHFR* gene, and MTHFR activity is closely related to *MTHFR* gene polymorphisms. The most important mutation of the *MTHFR* gene is C677T (SNP rs1801133 and Ala222Val), which can reduce MTHFR activity and produce heat intolerance [29]. The relationship between the *MTHFR* gene polymorphism and hypertension remains controversial [30]. In the present study, the relationship between *APOE* and *MTHFR* polymorphisms and hypertension was analyzed in a Hakka population.

## 2. Materials and Methods

### 2.1. Subjects

Between April 2016 and December 2020, 2,850 consecutive inpatients with clinically diagnosed hypertension and 2,034 non-hypertensive controls were retrospectively recruited from Meizhou People's Hospital in China. Inclusive criteria for hypertensive patients were the following: (1) Clinically diagnosed with hypertension. (2) Age ≥16 years old. Age, sex, smoking history, alcoholism history, medical history, and serum lipid levels of each subject were recorded. The control group consisted of healthy people who did not have hypertension.

### 2.2. DNA Extraction and Genotyping

The genomic DNA was extracted from blood samples using a DNA Blood Mini Kit (Qiagen GmbH, Germany). *APOE-* and *MTHFR-*related polymorphisms were amplified by polymerase chain reaction (PCR). The *APOE* gene was amplified using PCR at 50°C for 2 minutes, 95°C for 15 minutes, and 45 thermal cycles (94°C for 30 s and 65°C for 45 s) (Sinochips Bioscience Co., Ltd., Zhuhai, Guangdong, China). The *MTHFR* gene was amplified using PCR at 94°C for 5 minutes, followed by 35 thermal cycles (94°C for 25 s, 56°C for 25 s, and 72°C for 25 s) (BaiO Technology Co, Ltd, Shanghai, China). The PCR products were hybridized with wild-type or mutant probes fixed on the chip, and the genotypes of the samples were determined by the hybridization reaction.

### 2.3. Serum Lipid and Plasma Catecholamine Measurements

Serum lipid levels of the samples were evaluated by an Olympus AU5400 system (Olympus Corporation, Tokyo, Japan). Total cholesterol (TC), triglyceride (TG), low-density lipoprotein cholesterol (LDL-C), high-density lipoprotein cholesterol (HDL-C), apolipoprotein A1 (Apo-A1), and apolipoprotein B (Apo-B) analyses were performed. Enzyme-linked immunosorbent assay (ELISA) kits (Elabscience Biotechnology Co., Ltd, Wuhan, China) were used for evaluating the concentrations of epinephrine, norepinephrine, and dopamine.

### 2.4. Statistical Analysis

The SPSS statistical software version 21.0 (IBM Inc., USA) was used for data analysis. The measurement data were compared using either Student's *t*-test or the Mann–Whitney *U* test. Genotype composition ratios and allele frequencies between groups were analyzed by the Chi-square test. Logistic regression analysis was applied to examine the relationship between gene polymorphisms and hypertension.

## 3. Results

### 3.1. Characteristics of Subjects

This study included 4,884 participants. They were included in this study. 2,850 hypertensive patients (1,761 are men and 1,089 are women) and 2,034 controls (1,399 are men and 635 are women) were enrolled. The average age was 67.62 ± 11.76 years and 65.39 ± 13.04 years in hypertensive patients and controls, respectively. There were statistically significant differences in the percentage of subjects with a history of smoking (*P* < 0.001) and the percentage of subjects with a history of alcoholism (*P* < 0.001). The Hcy (*P* < 0.001), TG (*P*=0.008), TC (*P* < 0.001), LDL-C (*P*=0.001), Apo-A1 (*P* < 0.001), and Apo-B (*P* < 0.001) levels in the hypertensive subjects were higher than those in the controls (Table 1).

### 3.2. Frequencies of APOE rs429358, rs7412, and MTHFR rs1801133 Genotypes and Alleles in Hypertensive Patients and Controls

The frequencies of *APOE* rs429358, rs7412, and *MTHFR* rs1801133 genotypes and alleles were compared between hypertensive patients and nonhypertensive controls. The genotype distributions of *APOE* rs429358, rs7412, and *MTHFR* rs1801133 in hypertensive patients (*χ*^2^ = 0.389, *P*=0.533; *χ*^2^ = 3.340, *P*=0.068 and *χ*^2^ = 0.030, *P*=0.863) and controls (*χ*^2^ = 0.412, *P*=0.521; *χ*^2^ = 1.683, *P*=0.195 and *χ*^2^ = 0.0002, *P*=0.988) were consistent with Hardy–Weinberg equilibrium, respectively. It was found that there was no significant difference in the distribution of genotypes and alleles of *APOE* rs429358 and rs7412 between hypertensive patients and controls (*P* > 0.05). The same result was observed in the *MTHFR* rs1801133 gene (Table 2).

### 3.3. Characteristics of Hypertensive Patients Stratified by APOE rs429358 and rs7412 Genotypes, APOE *ɛ*2, *ɛ*3, and *ɛ*4 Alleles, and MTHFR Variants

The differences in characteristics in hypertensive patients stratified by *APOE* and *MTHFR* genotypes and alleles were analyzed. Patients with hypertension who were *APOE* rs429358 C/C homozygous had higher TG levels (2.09 ± 2.12 mmol/L vs. 1.71 ± 1.60 mmol/L in T/T and 1.99 ± 2.18 mmol/L in T/C, *P* < 0.001), higher TC levels (5.20 ± 1.38 mmol/L vs. 4.85 ± 1.40 mmol/L in T/T and 5.01 ± 1.44 mmol/L in T/C, *P*=0.003), higher LDL-C levels (3.01 ± 1.02 mmol/L vs. 2.69 ± 0.97 mmol/L in T/T and 2.88 ± 1.01 mmol/L in T/C, *P* < 0.001), and higher Apo-B levels (0.94 ± 0.34 g/L vs. 0.84 ± 0.28 g/L in T/T and 0.89 ± 0.31 g/L in T/C, *P* < 0.001), while individuals with the *APOE* rs429358T/T genotype had higher HDL-C levels (1.28 ± 0.39 mmol/L vs. 1.21 ± 0.39 mmol/L in T/C and 1.23 ± 0.32 mmol/L in C/C, *P* < 0.001) (Table 3).

Patients with hypertension who were *APOE* rs7412T/T homozygous had higher TG levels (2.74 ± 2.36 mmol/L vs. 1.76 ± 1.73 mmol/L in C/C and 1.74 ± 1.64 mmol/L in C/T, *P*=0.013), higher TC levels (5.24 ± 2.07 mmol/L vs. 4.93 ± 1.41 mmol/L in C/C and 4.54 ± 1.35 mmol/L in C/T, *P* < 0.001), lower LDL-C levels (2.11 ± 0.79 mmol/L vs. 2.78 ± 0.99 mmol/L in C/C and 2.38 ± 0.89 mmol/L in C/T, *P* < 0.001), and lower Apo-B levels (0.67 ± 0.25 g/L vs. 0.86 ± 0.29 g/L in C/C and 0.76 ± 0.27 g/L in C/T, *P* < 0.001) (Table 3).

Subjects with the *ε*2/*ε*4 genotype (*n* = 75, 45 patients and 30 controls) were excluded from the analysis of the relationship between *APOE* alleles and lipid levels because of the opposite effects of the *ε*2 and *ε*4 alleles. Hypertensive patients with the *APOE ɛ*4 allele had higher TG, TC, LDL-C, and Apo-B levels, and lower HDL-C and Apo-A1 levels (Table 3).

Hcy levels in patients with *MTHFR* CC, CT, and TT genotypes were increased (15.85 ± 6.59 *μ*mol/L, 16.76 ± 8.18 *μ*mol/L, and 21.74 ± 13.71 *μ*mol/L) (*P* < 0.001), while hypertensive patients with the TT genotype and T allele (21.74 ± 13.71 *μ*mol/L and 17.48 ± 9.35 *μ*mol/L) had higher Hcy levels than patients with other genotypes and the C allele (15.85 ± 6.59, 16.76 ± 8.18 *μ*mol/L, and 16.22 ± 7.29 *μ*mol/L) (*P* < 0.001) (Table 4).

### 3.4. Association of APOE rs429358, rs7412, and MTHFR rs1801133 Polymorphisms with Hypertension

The association between *APOE* rs429358 genotypes and hypertension was studied using three genetic modes: co-dominant mode (T/C vs. T/T, C/C vs. T/T), dominant mode (T/C plus C/C vs. T/T), and recessive mode (C/C vs. T/T plus T/C). The *APOE* rs429358 polymorphism in these genetic modes (gender-, age-, smoking-, and drinking-adjusted) was not a significant risk factor for hypertension. In the same way, the *APOE* rs7412T/T genotype in the co-dominant mode (T/T vs. C/C) (adjusted OR 2.682, 95% CI 1.072–6.710, *P*=0.035) was a significant risk factor for hypertension, while the *MTHFR* rs1801133 polymorphism in these genetic modes was not a significant risk factor for hypertension (Table 5).

### 3.5. Comparison of the Plasma Concentration of Catecholamines in Individuals with C/C, C/T, and T/T Genotypes of APOE rs7412 SNP

**Figure 1:**
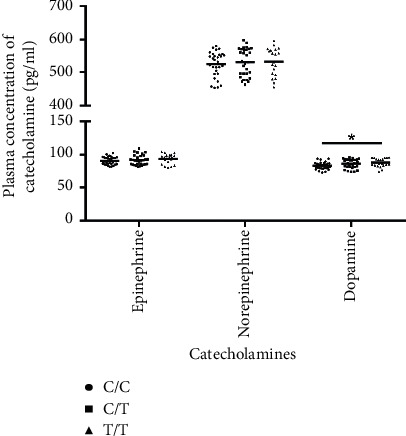
Comparison of plasma concentration of catecholamines in individuals with C/C (*n* = 30), C/T (*n* = 30), and T/T (*n* = 20) genotypes of *APOE* rs7412 SNP.

The plasma concentrations of catecholamines in individuals with C/C (*n* = 30), C/T (*n* = 30), and T/T (*n* = 20) genotypes of *APOE* rs7412 SNP were compared. Individuals who were *APOE* rs7412T/T homozygous had higher dopamine levels (87.89 ± 6.67 pg/ml vs. 83.11 ± 5.50 pg/ml, *P*=0.011) than those with *APOE* rs7412C/C. Individuals who were *APOE* rs7412T/T homozygous had higher epinephrine levels (93.59 ± 7.60 pg/ml vs. 90.69 ± 6.09 pg/ml) and higher norepinephrine levels (532.97 ± 42.55 pg/ml vs. 524.96 ± 38.44 pg/ml) than those with *APOE* rs7412C/C, but the differences were not statistically significant (Figure 1).

## 4. Discussion

Hypertension is one of the leading causes of the global burden of some diseases [1]. Lipid levels have been linked to the risk of hypertension. Abnormal lipid metabolism caused by genetic factors is closely related to the incidence of cardiovascular and cerebrovascular diseases, with the *APOE* gene being one of the most important genes affecting lipid metabolism [31]. The serum Hcy level is linked to the incidence of hypertension. *MTHFR* gene polymorphisms are associated with MTHFR activity and Hcy metabolic disorder and cause hyperhomocysteinemia [29]. In this study, the association of *APOE* rs429358, rs7412, and *MTHFR* rs1801133 genetic polymorphisms with hypertension was analyzed in a Hakka population.

Hcy is an intermediate metabolite of the methionine cycle in the body. Hyperhomocysteinemia may increase the risk of some diseases, including hypertension, cardiovascular disorders, pulmonary embolism, and depression [32]. In the present study, hypertensive patients had significantly higher serum Hcy levels than nonhypertensive controls, implying that hyperhomocysteine may be involved in the pathogenesis of hypertension. MTHFR is an enzyme involved in homocysteine metabolism. The gene that encodes this enzyme has many gene polymorphisms, and the most studied polymorphism is *MTHFR* rs1801133 (C677T). It has been shown that the homozygous (TT) genotype of the *MTHFR* rs1801133 polymorphism has higher plasma Hcy levels than the heterozygous (CT) and wild (CC) genotypes [33].

So far, there have been many studies on the relationship between *MTHFR* rs1801133 polymorphism and hypertension. It has been reported that there is no correlation between the *MTHFR* polymorphism and hypertension in Japanese [34, 35], Chinese [36], Danish [37], and Caucasians [38]. On the contrary, the *MTHFR* rs1801133C/T genotype was a risk factor for hypertension in a Caucasian population [39]. People who carried the *MTHFR* rs1801133T allele had a higher risk of hypertension among Chinese in Taiwan [40], a Chinese Han population in Shihezi city [41], Chinese from Jiangxi Province [42], Argentineans from Buenos Aires city [43, 44], and Spaniards [45]. Based on our findings, the *MTHFR* rs1801133 polymorphism was not associated with an increased risk of hypertension in the Hakka population, but this needs to be confirmed with a larger sample size.

There have been a few studies on the relationship between *APOE* polymorphisms and hypertension, and these studies have shown inconsistent results. Studies have found that people who carry the *ε*4 allele have an increased risk of hypertension [46–48]. The *APOE* gene was not related to the risk of hypertension [49]. In addition to the common *APOE* polymorphisms, other polymorphisms of *APOE* have also been reported in this relationship. *APOE* + 2836G>A polymorphism is associated with the susceptibility to hypertension [50]. An animal study has shown that the pathogenesis of hypertension in ApoE(−/−)/Cyp1b1(+/+) mice fed a high-fat diet is most likely due to oxidative stress caused by CYP1B1, as well as increased plasma lipid levels [51]. The *APOE* rs7412T/T genotype was found to be a risk factor for hypertension in this study. It has been reported that hypertensive patients have higher catecholamine levels [52, 53]. The results of this study showed that *APOE* rs7412T/T subjects had higher catecholamine levels than C/C subjects, but there was no statistically significant difference. The pathophysiology of hypertension may be influenced by functional changes in ApoE, which regulates lipoprotein metabolism, as well as sympathetic nervous excitement manifested by elevated plasma catecholamine levels.

The inconsistent results of the above studies may be due to differences in race and sample size. This study is the first to investigate the relationship between both *APOE* and *MTHFR* gene polymorphisms and hypertension in the Hakka people. This research has some limitations. First and foremost, information about the subjects' Hakka ethnic characteristics was obtained solely through their own descriptions, with no genetic information analysis. Second, because this is a retrospective study of patients in medical institutions and physical examination subjects, there may be some selection bias because the population is not fully representative. Third, the relationship between only the common SNPs of *APOE* and *MTHFR* and hypertension was analyzed, but other polymorphisms of *APOE* and *MTHFR* may also influence the development of hypertension. In the future, larger sample sizes, more genes, and polymorphisms will be required to investigate this relationship.

## 5. Conclusion

In summary, the *APOE* rs7412T/T genotype may be a risk factor for hypertension in the Chinese Hakka population. It provides evidence that *APOE* gene polymorphisms are linked to hypertension.

## Figures and Tables

**Table 1:** Clinical characteristics of hypertensive patients and control participants.

	Total (*n* = 4,884)	Hypertensive patients (*n* = 2,850)	Controls (*n* = 2,034)	*P* values
Age, years	66.69 ± 12.36	67.62 ± 11.76	65.39 ± 13.04	<0.001

Gender				
Male, *n*(%)	3160 (64.70)	1761 (61.79)	1399 (68.78)	<0.001
Female, *n*(%)	1724 (35.30)	1089 (38.21)	635 (31.22)	
History of smoking, *n*(%)	1361 (27.87)	694 (24.35)	667 (32.79)	<0.001
History of alcoholism, *n*(%)	309 (6.33)	132 (4.63)	177 (8.70)	<0.001
SBP, mmHg	141.78 ± 27.02	150.96 ± 25.67	128.92 ± 23.38	<0.001
DBP, mmHg	82.55 ± 15.64	86.27 ± 15.56	77.35 ± 14.22	<0.001
Hcy, *μ*mol/L	16.57 ± 7.97	16.95 ± 7.96	16.04 ± 7.97	<0.001
TG, mmol/L	1.77 ± 1.73	1.82 ± 1.65	1.69 ± 1.82	0.008
TC, mmol/L	4.88 ± 1.41	4.95 ± 1.34	4.78 ± 1.50	<0.001
HDL-C, mmol/L	1.27 ± 0.39	1.28 ± 0.38	1.25 ± 0.41	0.015
LDL-C, mmol/L	2.72 ± 0.98	2.77 ± 0.95	2.67 ± 1.03	0.001
Apo-A1, g/L	1.10 ± 0.33	1.13 ± 0.32	1.07 ± 0.34	<0.001
Apo-B, g/L	0.85 ± 0.29	0.86 ± 0.28	0.82 ± 0.30	<0.001

**Table 2:** Frequencies of *APOE* rs429358, rs7412, and *MTHFR* rs1801133 genotypes and alleles in hypertensive patients and controls.

	Genotype/allele	Hypertensive patients (*n* = 2,850)	Controls (*n* = 2,034)	*χ * ^2^	*P* value
*APOE* rs429358					
	T/T	2320 (81.40%)	1633 (80.29%)	0.985	0.609
	T/C	506 (17.75%)	382 (18.78%)		
	C/C	24 (0.84%)	19 (0.93%)		
	T	5146 (90.28%)	3648 (89.68%)	0.968	0.338
	C	554 (9.72%)	420 (10.32%)		
	HWE (*χ*^2^, *P*)	*χ * ^2^ = 0.389, *P*=0.533	*χ * ^2^ = 0.412, *P*=0.521		

*APOE* rs7412					
	C/C	2464 (86.46%)	1758 (86.43%)	4.391	0.111
	C/T	365 (12.81%)	270 (13.27%)		
	T/T	21 (0.74%)	6 (0.29%)		
	C	5293 (92.86%)	3786 (93.07%)	0.157	0.718
	T	407 (7.14%)	282 (6.93%)		
	HWE (*χ*^2^, *P*)	*χ * ^2^ = 3.340, *P* = 0.068	*χ * ^2^ = 1.683, *P*=0.195		

*MTHFR* rs1801133					
	C/C	1573 (55.19%)	1145 (56.29%)	0.615	0.736
	C/T	1091 (38.28%)	762 (37.46%)		
	T/T	186 (6.53%)	127 (6.24%)		
	C	4237 (74.33%)	3052 (75.02%)	0.599	0.450
	T	1463 (25.67%)	1016 (24.98%)		
	HWE (*χ*^2^, *P*)	*χ * ^2^ = 0.030, *P*=0.863	*χ * ^2^ = 0.0002, *P*=0.988		

HWE, Hardy Weinberg equilibrium.

**Table 3:** Clinical characteristics of subjects stratified by *APOE* rs429358 and rs7412 genotypes and ɛ2, ɛ3, and ɛ4 alleles.

Clinical characteristics	rs429358				rs7412				*APOE* alleles			
	T/T (*n* = 3,953)	T/C (*n* = 888)	C/C (*n* = 43)	T/C+ C/C (*n* = 931)	C/C (*n* = 4,222)	C/T (*n* = 635)	T/T (*n* = 27)	C/T+ T/T (*n* = 662)	*ɛ*2 (*n* = 587)	*ɛ*3 (*n* = 3,366)	*ɛ*4 (*n* = 856)	*P*values
Age, years	66.92 ± 12.39	65.80 ± 12.20^*∗*^	64.37 ± 12.04	65.73 ± 12.19^*∗∗*^	66.67 ± 12.27	66.72 ± 12.83	68.67 ± 14.47	66.80 ± 12.89	67.11 ± 12.58	66.88 ± 12.35	65.85 ± 11.91	0.067
Gender												
Male, *n*(%)	2547 (64.43)	583 (65.65)	30 (69.77)	613 (65.84)	2738 (64.85)	400 (62.99)	22 (81.48)	422 (63.75)	367 (62.52)	2180 (64.77)	558 (65.19)	0.529
Female, *n*(%)	1406 (35.57)	305 (34.35)	13 (30.23)	318 (34.16)	1484 (35.15)	235 (37.01)	5 (18.52)	240 (36.25)	220 (37.48)	1186 (35.23)	298 (34.81)	
History of smoking, *n*(%)	1101 (27.85)	247 (27.82)	13 (30.23)	260 (27.93)	1198 (28.38)	153 (24.09)^*∗*^	10 (37.04)	163 (24.62)^*∗*^	146 (24.87)	955 (28.37)	243 (28.39)	0.209
History of alcoholism, *n*(%)	256 (6.48)	52 (5.86)	1 (2.33)	53 (5.69)	267 (6.32)	40 (6.30)	2 (7.41)	42 (6.34)	39 (6.64)	217 (6.45)	50 (5.84)	0.771
SBP, mmHg	142.09 ± 26.98	140.61 ± 27.38	138.23 ± 22.86	140.50 ± 27.18	141.81 ± 26.79	141.54 ± 28.53	142.96 ± 27.36	141.60 ± 28.47	141.53 ± 28.44	142.18 ± 26.72	140.36 ± 27.05	0.204
DBP, mmHg	82.69 ± 15.58	81.90 ± 15.85	83.51 ± 17.02	81.98 ± 15.90	82.62 ± 15.54	82.08 ± 16.35	83.81 ± 14.60	82.15 ± 16.27	82.18 ± 16.40	82.78 ± 15.43	81.98 ± 15.95	0.337
Hcy, *μ*mol/L	16.64 ± 8.08	16.33 ± 7.57	15.27 ± 5.16	16.28 ± 7.48	16.59 ± 7.87	16.44 ± 8.72	17.56 ± 4.88	16.48 ± 8.60	16.31 ± 7.62	16.70 ± 8.16	16.15 ± 6.58	0.133
TG, mmol/L	1.71 ± 1.60	1.99 ± 2.18^*∗∗*^	2.09 ± 2.12	2.00 ± 2.18^*∗∗*^	1.76 ± 1.73	1.74 ± 1.64	2.74 ± 2.36^*∗*^	1.79 ± 1.69	1.75 ± 1.70	1.71 ± 1.58	2.00 ± 2.22	<0.001
TC, mmol/L	4.85 ± 1.40	5.01 ± 1.44^*∗∗*^	5.20 ± 1.38	5.02 ± 1.44^*∗∗*^	4.93 ± 1.41	4.54 ± 1.35^*∗∗*^	5.24 ± 2.07	4.57 ± 1.39^*∗∗*^	4.57 ± 1.43	4.90 ± 1.39	5.06 ± 1.46	<0.001
HDL-C, mmol/L	1.28 ± 0.39	1.21 ± 0.39^*∗∗*^	1.23 ± 0.32	1.21 ± 0.38^*∗∗*^	1.26 ± 0.39	1.28 ± 0.39	1.27 ± 0.42	1.28 ± 0.39	1.29 ± 0.39	1.28 ± 0.40	1.21 ± 0.38	<0.001
LDL-C, mmol/L	2.69 ± 0.97	2.88 ± 1.01^*∗∗*^	3.01 ± 1.02^*∗*^	2.88 ± 1.01^*∗∗*^	2.78 ± 0.99	2.38 ± 0.89^*∗∗*^	2.11 ± 0.79^*∗∗*^	2.37 ± 0.89^*∗∗*^	2.35 ± 0.90	2.75 ± 0.97	2.92 ± 1.03	<0.001
Apo-A1, g/L	1.12 ± 0.33	1.06 ± 0.33^*∗∗*^	1.07 ± 0.28	1.06 ± 0.33^*∗∗*^	1.10 ± 0.33	1.12 ± 0.33	1.12 ± 0.26	1.12 ± 0.32	1.13 ± 0.32	1.11 ± 0.33	1.06 ± 0.33	<0.001
Apo-B, g/L	0.84 ± 0.28	0.89 ± 0.31^*∗∗*^	0.94 ± 0.34^*∗*^	0.89 ± 0.31^*∗∗*^	0.86 ± 0.29	0.76 ± 0.27^*∗∗*^	0.67 ± 0.25^*∗∗*^	0.76 ± 0.27^*∗∗*^	0.75 ± 0.27	0.85 ± 0.28	0.90 ± 0.31	<0.001

Compared to the patients with the wild-type genotype, ^*∗*^*P* < 0.05 and ^*∗∗*^*P* < 0.01 for rs429358 and rs7412.

**Table 4:** Clinical characteristics of subjects stratified by *MTHFR* rs1801133 genotypes and alleles.

Clinical characteristics	C/C (*n* = 2,718)	C/T (*n* = 1,853)	T/T (*n* = 313)	*P* values	C allele (C/C + C/T) (*n* = 4,571)	T allele (C/T + T/T) (*n* = 2,166)	*P* values
Age, years	66.81 ± 12.38	66.66 ± 12.31	65.81 ± 12.40	0.394	66.75 ± 12.35	66.54 ± 12.32	0.505

Gender							
Male, *n*(%)	1753 (64.50)	1193 (64.38)	214 (68.37)	0.377	2946 (64.45)	1407 (64.96)	0.703
Female, *n*(%)	965 (35.50)	660 (35.62)	99 (31.63)		1625 (35.55)	759 (35.04)	
History of smoking, *n*(%)	752 (27.67)	520 (28.06)	89 (28.43)	0.933	1272 (27.83)	609 (28.12)	0.816
History of alcoholism, *n*(%)	170 (6.25)	119 (6.42)	20 (6.39)	0.977	289 (6.32)	139 (6.42)	0.915
SBP, mmHg	141.80 ± 27.63	141.73 ± 26.68	141.99 ± 23.52	0.987	141.77 ± 27.25	141.77 ± 26.25	0.999
DBP, mmHg	82.23 ± 15.69	82.83 ± 15.73	83.67 ± 14.62	0.190	82.48 ± 15.71	82.95 ± 15.57	0.243
Hcy, *μ*mol/L	15.85 ± 6.59	16.76 ± 8.18	21.74 ± 13.71	<0.001	16.22 ± 7.29	17.48 ± 9.35	<0.001
TG, mmol/L	1.77 ± 1.85	1.76 ± 1.58	1.73 ± 1.51	0.904	1.77 ± 1.74	1.76 ± 1.57	0.781
TC, mmol/L	4.91 ± 1.44	4.84 ± 1.38	4.89 ± 1.30	0.301	4.88 ± 1.42	4.85 ± 1.37	0.366
HDL-C, mmol/L	1.27 ± 0.40	1.26 ± 0.39	1.26 ± 0.35	0.595	1.27 ± 0.40	1.26 ± 0.38	0.518
LDL-C, mmol/L	2.74 ± 0.99	2.71 ± 0.98	2.74 ± 0.95	0.536	2.72 ± 0.99	2.71 ± 0.97	0.609
Apo-A1, g/L	1.11 ± 0.34	1.10 ± 0.33	1.11 ± 0.29	0.719	1.10 ± 0.33	1.10 ± 0.33	0.819
Apo-B, g/L	0.85 ± 0.29	0.84 ± 0.29	0.86 ± 0.29	0.678	0.85 ± 0.29	0.85 ± 0.29	0.904

**Table 5:** Association of *APOE* rs429358, rs7412, and *MTHFR* rs1801133 polymorphisms with hypertension.

SNP	Model	Genotype	Hypertension (*n* = 2,850)	Control (*n* = 2,034)	Univariate OR (95% CI)	*P* values	Multivariate OR (95% CI)	*P* values
*APOE* rs429358								
	Co-dominant	T/T	2320 (81.40%)	1633 (80.29%)	1.000 (reference)			
		T/C	506 (17.75%)	382 (18.78%)	0.932 (0.805–1.080)	0.351	0.929 (0.801–1.078)	0.333
		C/C	24 (0.84%)	19 (0.93%)	0.889 (0.485–1.628)	0.703	0.901 (0.490–1.657)	0.738
	Dominant	T/T	2320 (81.40%)	1633 (80.29%)				
		T/C + C/C	530 (18.60%)	401 (19.71%)	0.930 (0.805–1.075)	0.327	0.928 (0.802–1.074)	0.316
	Recessive	T/T + T/C	2826 (99.16%)	2015 (99.07%)				
		C/C	24 (0.84%)	19 (0.93%)	0.901 (0.492–1.649)	0.734	0.918 (0.500–1.689)	0.784

*APOE* rs7412								
	Co-dominant	C/C	2464 (86.46%)	1758 (86.43%)	1.000 (reference)			
		C/T	365 (12.81%)	270 (13.27%)	0.965 (0.815–1.142)	0.675	0.950 (0.802–1.127)	0.559
		T/T	21 (0.74%)	6 (0.29%)	2.497 (1.006–6.200)	0.049	2.682 (1.072–6.710)	0.035
	Dominant	C/C	2464 (86.46%)	1758 (86.43%)	1.000 (reference)			
		C/T + T/T	386 (13.54%)	276 (13.57%)	0.998 (0.845–1.178)	0.980	0.987 (0.834–1.167)	0.874
	Recessive	C/C + C/T	2829 (99.26%)	2028 (99.71%)	1.000 (reference)			
		T/T	21 (0.74%)	6 (0.29%)	2.509 (1.011–6.227)	0.047	2.689 (1.075–6.729)	0.034

*APOE* allele								
		*ɛ*2 carrier^a^	341 (11.96%)	246 (12.09%)	0.989 (0.830–1.178)	0.901	0.976 (0.818–1.164)	0.783
		*ɛ*4 carrier^b^	485 (17.02%)	371 (18.24%)	0.920 (0.793–1.068)	0.275	0.917 (0.789–1.066)	0.260

*MTHFR* rs1801133								
	Co-dominant	C/C	1573 (55.19%)	1145 (56.29%)	1.000 (reference)			
		C/T	1091 (38.28%)	762 (37.46%)	1.042 (0.924–1.175)	0.499	1.048 (0.929–1.183)	0.444
		T/T	186 (6.53%)	127 (6.24%)	1.066 (0.840–1.353)	0.598	1.077 (0.847–1.369)	0.545
	Dominant	C/C	1573 (55.19%)	1145 (56.29%)	1.000 (reference)			
		C/T + T/T	1277 (44.81%)	889 (43.71%)	1.046 (0.932–1.173)	0.446	1.052 (0.938–1.181)	0.386
	Recessive	C/C + C/T	2664 (93.47%)	1907 (93.76%)	1.000 (reference)			
		T/T	186 (6.53%)	127 (6.24%)	1.048 (0.830–1.324)	0.691	1.060 (0.837–1.341)	0.630

^a^ *ɛ*2/*ɛ*2 plus *ɛ*2/*ɛ*3, reference genotype: *ɛ*3/*ɛ*3 plus *ɛ*3/*ɛ*4 plus *ɛ*4/*ɛ*4. ^b^*ɛ*3/*ɛ*4 plus *ɛ*4/*ɛ*4, reference genotype: *ɛ*2/*ɛ*2 plus *ɛ*2/*ɛ*3 plus *ɛ*3/*ɛ*3.

## Data Availability

All data reported are included in the manuscript. Any clarification and additional requests can be made to the corresponding author.
